# The emerging mechanism behind viral infections and extracellular vesicles hypotheses leading to neuroinflammation and Alzheimer's disease pathology

**DOI:** 10.1002/ibra.12090

**Published:** 2023-01-31

**Authors:** Mai M. Anwar

**Affiliations:** ^1^ Department of Biochemistry National Organization for Drug Control and Research (NODCAR)/Egyptian Drug Authority (EDA) Cairo Egypt

**Keywords:** Alzheimer's disease, Aβ, CNS infection, herpes simplex virus (HSV), neuroinflammation, varicella‐zoster virus (VZV)

## Abstract

Despite decades of repeated and intense research, the etiology of sudden Alzheimer's disease (AD) symptoms is still unclear. AD progressive pathology mainly involves neuron damage, depositions of amyloid‐beta (Aβ), and hyperphosphorylated tau protein. All these defects are manifested by exaggerated cytokine storm and neuroinflammation leading to irreversible brain damage in the long term. Despite the numerous risks and drawbacks associated with AD, it is believed that there is a hidden unknown causative and predisposing factors for AD. Extracellular vesicles (EVs) are small vesicles released by cells as a type of intercellular communication. Several pieces of evidence support the inclusion of viral components within EVs facilitating their penetration into the blood–brain barrier leading to neuroinflammation. In light of the SARS‐CoV‐19 pandemic and its related neurological complications, it is mandatory to highlight the possibility and viability of viral infections such as varicella‐zoster virus (VZV) and herpes simplex virus (HSV) on the onset of AD. Herein, the author is investigating the potential role of VZV and HSV along with highlighting the suggested route of pathogenesis entry resulting in AD manifestations. Additionally, this review aims to summarize the role of EVs in mediating the central nervous system viral infections leading to AD.

## Graphical abstract

In the current study, we are highlighting the emerging mechanism of viral infections in Alzheimer's disease. Once infiltrated viral infection reaches the central nervous system via penetrating the blood–brain barrier, it directly results in a direct shift from resting microglia phenotype to reactive M1 microglia phenotype. The activation of microglia M1 phenotype cells triggers the release of inflammatory cytokines and mediators resulting in severe brain damage. This leads to exaggerated neurotoxins formation which results in neuroinflammation ending with tau protein and Aβ plaque depositions. Thus, viral infection may be considered a key pathogenic predisposing factor in the development of sudden Alzheimer's disease.

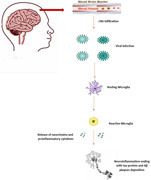

## 1 INTRODUCTION

Dementia is a progressive decline of brain functionality, including cognitive impairments, behavior changes, mood disturbance, and imbalance. All these manifestations have been related to aging and Alzheimer's disease (AD). AD is currently the most common type of dementia, affecting more than 60 million people worldwide with an expected increase in the coming years.^1^, ^2^ Symptoms associated with AD and its related disorders are mainly due to hallmarks deposition including extracellular amyloid‐beta (Aβ) aggregations and intracellular tau proteins ending with severe brain damage.^3^ Currently, the most known underlying AD causes and hypotheses are unregulated Aβ cascade, microgliosis, neuroinflammation, and viral infections.^4^, ^5^ In light of the recent COVID‐19 pandemic and its related neurological complications,^6^, ^7^ viral infection hypothesis‐related AD complications have gained a lot of attention where the idea that a viral infection can act as a predisposing factor for AD goes back to.^8^ Ball et al.^8^ also highlighted that viral infections including herpes simplex virus (HSV) and varicella‐zoster virus (VZV) may play a vital role in the development and progression of AD. The suggested mechanism underlying AD‐viral pathogenicity mainly includes direct and indirect pathways. Accordingly, the direct infective pathway includes the direct invasive entry of the virus to the brain leading to severe cytokine storm and inflammation ending with neuron damage and AD pathological hallmarks.^9^ On the other hand, the indirect viral infective pathway can be defined by the indirect entry of the virus to the brain where the AD pathology is suggested to be mediated by the indirect‐induced systemic inflammation.^10^, ^11^ On both sides, inflammation is the main driving predisposing factor for the underlying AD pathology‐related viral infection. Additionally, it is suggested that extracellular vesicles (EVs) can be involved in AD pathology‐related viral infection where EVs can act as transferable vectors to misfolded proteins.^12^, ^13^ Herein, the review is highlighting the possibility of VZV and HSV in the involvement of cognitive dysfunctions and AD pathogenesis. The suggested route of penetration, clinical manifestations, EVs involvements, and postmortem findings are discussed to elaborate and highlight the severity of viral infections in the brain.

## 2 THE ROLE OF MICROGLIA CELLS UNDER THE CONDITION OF VIRAL INFECTIONS

Microglia cells are the resident phagocytes macrophage type within the brain which act as the first shield against pathogenesis and inflammation.^5^, ^14^ During healthy homeostatic balanced conditions, microglia cells support neuronal functions and maintain connectivity.^14^, ^15^ Additionally, the main roles of microglia cells also involve maintaining myelin homeostasis, synaptic pruning, and tissue surveillance.^5^, ^14^ Upon instant viral invasion, microglia cells were found to be heavily activated and shifted from the balanced homeostatic phase to their enlarged cell body phenotype with various retracted cell processes.^16^, ^17^ To find out their exact roles during infection, the effects of microglia pharmaceutical depletion under viral infections were studied. Upon microglial depletion, a significant increase in the mortality rate of the central nervous system (CNS)‐infected models was reported.^14^ Furthermore, viral loads within the infected CNS models were highly enhanced under conditions of microglial cell depletion. Under such conditions of induced microglial cell depletion in mouse models, the development of seizures, increase viral infection loads, hippocampal neuron degeneration, and cognitive dysfunctions were observed.^18^ This highlights the importance and protective actions of microglia against viral infections.

## 3 DIRECT AND INDIRECT VIRAL PATHOGENIC PATHWAYS FOR VZV AND HSV LEADING TO AD PATHOLOGY

**Figure 1:**
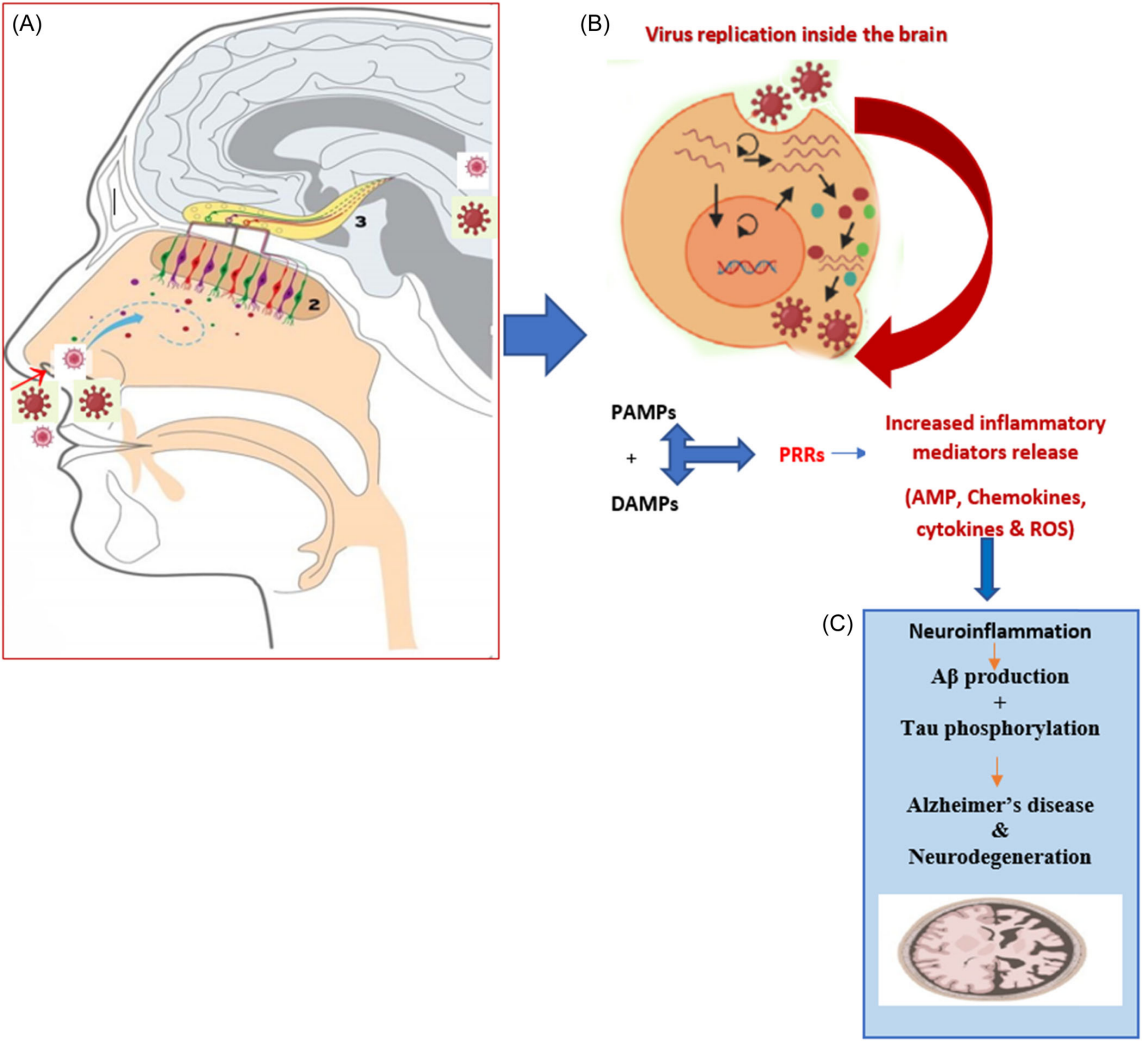
Illustration of HSV‐1 and varicella‐zoster virus (VZV) direct route of penetration into the brain. (A) The main direct suggested route of penetration is via the olfactory bulb and nasal cavity infecting the epithelial cell line and causing a cold sore. (B) Once the viral infection reaches the brain it directly starts to replicate, resulting in the release of pathogen‐associated molecular patterns (PAMPs) and damage‐associated molecular patterns (DAMPs). Both are then recognized by the pathogen recognition receptors (PRR) leading to the release of inflammatory mediators such as reactive oxygen species (ROS), cytokines, AMP, and chemokines. (C) These triggered and persistent inflammatory responses result in acute followed by chronic neuroinflammation resulting in Aβ and tau protein hallmarks deposition ending with AD and neurodegeneration _ENREF_6.^6^ [Color figure can be viewed at wileyonlinelibrary.com]

**Figure 2:**
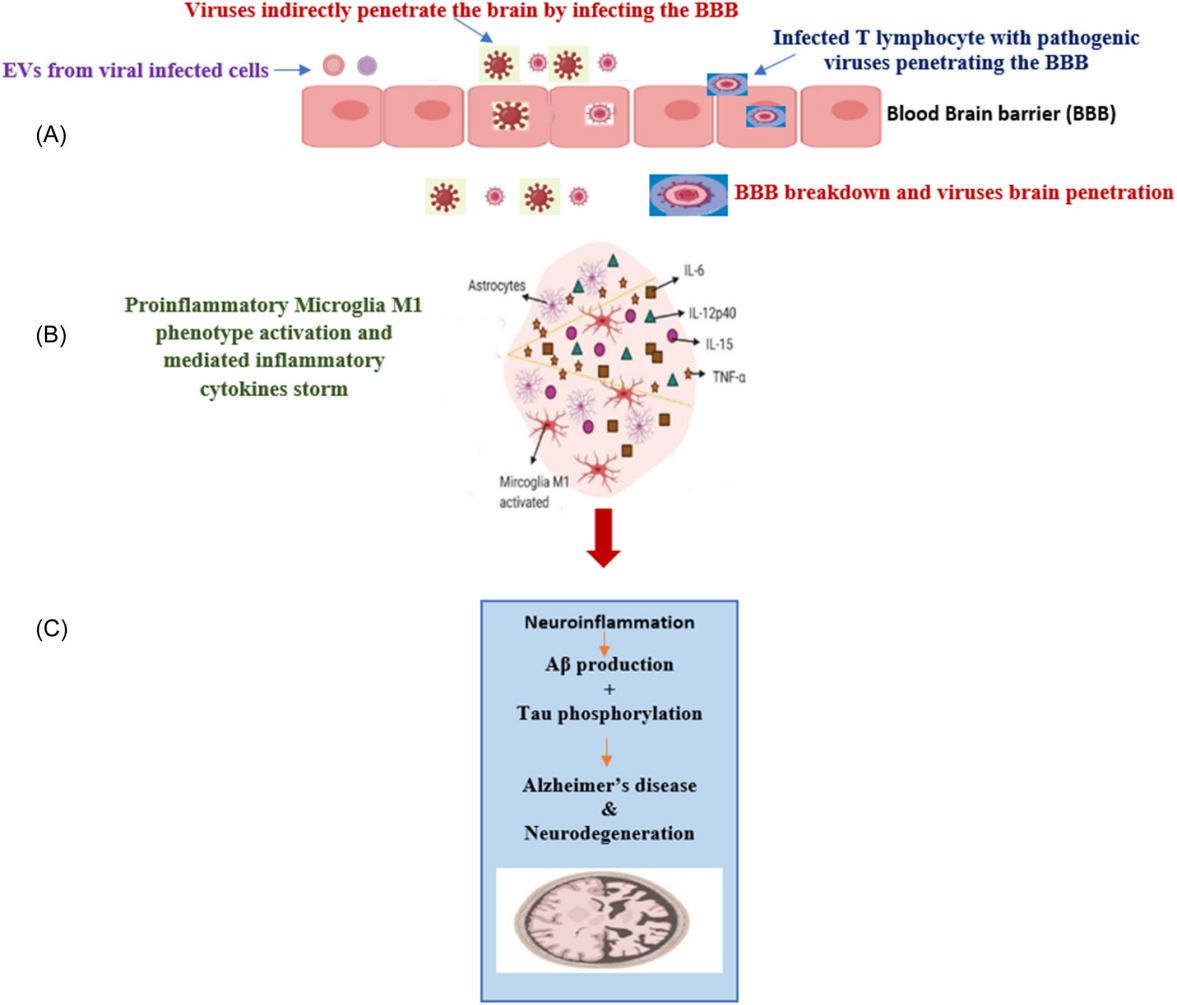
Illustration of indirect HSV‐1 and varicella‐zoster virus (VZV) infection to the brain via peripheral circulation. (A) Once the viral infection infects the peripheral circulation, it results in triggered inflammatory cytokines storms leading to the disruption of the blood–brain barrier (BBB). As a result of BBB disruption, viral infection can directly cross the brain. The virus may also use T lymphocytes or extracellular vesicles (EVs) to cross the BBB. (B) Once the virus crosses the BBB, it mediates the release of TNF‐α and interleukin (IL)‐6 leading to the activation of microglia M1 phenotype and astrocytes. Additionally, inflammatory mediators may also be carried in infected EVs from the peripheral circulation to the brain leading to the activation of microglia M1 phenotype and astrocytes. (C) These activated microglia M1 phenotypes and astrocytes along with triggered cytokines storms result in acute followed by chronic neuroinflammation resulting in Aβ and tau protein hallmarks deposition ending with Alzheimer's disease and neurodegeneration.^6^ [Color figure can be viewed at wileyonlinelibrary.com]

There are two suggested main routes for VZV and HSV to enter the brain whether via direct and/or indirect infective pathways. The direct infective pathways as illustrated in Figure 1 may involve the direct invasion of VZV and HSV to the oral or nasal mucosal epithelial cells, followed by the olfactory bulb. This results in the direct invasion of the immune system leading to wide systemic deteriorative effects including the peripheral nervous system (PNS), especially under the condition of stress, chemotherapy, and immunodeficiency. Additionally, both viruses can reach the CNS via sensory neuron routes which include pseudounipolar axons, synapses, and traversing epithelial cells in the same pathogenic manner as COVID‐19.^6^, ^19^ Secondly, VZV and HSV might be involved in entering the CNS indirectly via blood circulation as illustrated in Figure 2. Several preclinical studies have reported that HSV and VZV might reach different brain regions including the midbrain, cerebral cortex, and cerebellum via infected blood circulation. Thus, systemic infected blood circulation may result in exaggerated inflammatory responses, triggered lymphocytes, and the activation of cytokines storms which may lead to the disruption of BBB.^20^, ^21^ Both viruses can directly infiltrate the BBB via infected macrophages by stimulating the release of tumor necrosis factor‐α (TNF‐α) and interleukin‐1β (IL‐1β) leading to the activation of M1 pro‐inflammatory microglia phenotype resulting in the AD pathological hallmarks deposition in the brain on the long term.^22^

## 4 VARICELLA‐ZOSTER VIRUS (VZV) AND ALZHEIMER'S DISEASE

Varicella‐zoster virus (VZV), is a neurotropic alpha herpes virus type exclusively found among humans causing repeatedly wide different types of disorders throughout their lifetime. VZV is mainly caused during childhood by an acute type of febrile exanthematous illness named varicella or chickenpox.^23^, ^24^ When chickenpox resolves, VZV directly becomes latent and is found to spread to the cranial nerves, and dorsal root of ganglia in addition to the entire neuraxis.^23^, ^24^, ^25^ Additionally, elderly and immunocompromised patients may be subjected to reactivated VZV with a dermatome rash and severe persistent pain. Thereby, VZV infection is not only occurring in children but also in young and elderly subjects. Following two weeks from a viral infection, VZV was found to spread to the CNS and spinal cord in addition to being detected among CSF leading to severe neurological complications including cognitive decline, motor dysfunctions, and meningoencephalitis.^23^, ^24^, ^25^ All these neurological complications can be related to the exaggerated release of pro‐inflammatory cytokines storms, chemokines, TNF‐α, and nuclear factor kappa B (NF‐kB) leading to severe neuroinflammation and AD hallmarks deposition detected among CSF and brain tissues in postmortem findings.^25^, ^26^, ^27^

### 4.1 Clinical and postmortem findings

Herein, we are evaluating and highlighting cognitive impairments associated with VZV infection by detecting cognitive declines, behavior changes, and hallmarks deposition among brain tissues and CSF clinically and in postmortem findings. It was reported that VZV might be resident in brain tissues and upon exposure to any infection or stress, it becomes reactivated leading to AD pathology and its associated dysfunctions. This can be considered a drawback of severely driven neuroinflammation and microglia M1 activation.^28^ All these previously reported findings can also be due to the reactivation of HSV‐1 which is strongly implicated in AD.^28^ Additionally, it was reported that VZV doesn't directly result in AD hallmarks deposition among the brain and spinal cord but indirectly may lead to AD and its related cognitive decline due to exaggerated inflammatory cytokines release and gliosis.^28^ Meanwhile, Bernstein et al.^29^ reported three published studies from different independent groups which have highlighted that VZV can be considered a risk factor for AD and dementia. Additionally, it was also reported that repeated signs of cognitive impairment were detected among VZV‐infected patients including learning disabilities and memory decline along with motor and learning dysfunctions.^29^ On the other hand, it was also reported that the presence of insulin‐degrading enzymes may act as a predisposing factor for increasing the severity of VZV as well as for the development of AD.^29^, ^30^ A Swedish study reported that compared with healthy control subjects, 9‐14 patients who had previously been infected with VZV reported neurological and cognitive dysfunctions within three years after virus infection.^31^ Additionally, Hokkanen et al.^32^ highlighted that persistent cognitive symptoms including memory impairment, and dementia have been noticed in the regular neurological follow‐ups following VZV infection. Whereas, Bubak et al.^33^ indicated that VZV infection may result in amyloid β hallmarks deposition and may act as a predisposing factor for severe cytotoxicity and inflammation leading to disease progression. The degree of cognitive decline was also studied three years post‐VZV infection. It was also reported that viral infections may affect attention, speed, executive function, learning abilities, and memory in infected patients.^31^, ^34^

## 5 HERPES SIMPLEX VIRUS TYPE 1 (HSV‐1) AND ALZHEIMER'S DISEASE

Herpes simplex virus type 1 (HSV1) is the most strongly associated herpes virus type associated with AD pathology. Repeated studies demonstrated a highly increased risk of AD as a drawback of HSV‐1.^35^, ^36^, ^37^ It was observed that in‐vitro and in‐vivo HSV‐1 inoculation directly results in AD hallmarks deposition in the brain and spinal cord along with being detected in CSF.^36^, ^37^ HSV‐1 infection whether reached the CNS directly or indirectly was found to affect different brain regions resulting in severe neuroinflammation and leading eventually to progressive AD pathology. Once reaching the CNS, the virus directly begins to replicate along with the release of pathogen‐associated molecular patterns (PAMPs) and damage‐associated molecular patterns (DAMPs) leading to lipopolysaccharides (LPS) like effects.^35^, ^36^, ^37^, ^38^, ^39^, ^40^ Consequently, this results in the activation and release of pro‐inflammatory cytokines, chemokines, and reactive oxygen species (ROS) associated with TNF‐α and NF‐kB. Ultimately, wide exaggerated immune responses against HSV‐1 are characterized by aggressive inflammatory responses and the activation of M1 microglia type leading to AD.^35^, ^36^, ^37^, ^41^, ^42^ Meanwhile, it was also suggested that glycoprotein B (GB) located in HSV‐1 produces a series of β‐pleated sheets nearly resembling Aβ fibrils acting as a predisposing factor for toxic Aβ depositions into cortical neurons.^43^ On the other hand, HSV‐1 was found to increase the hyperphosphorylation of tau protein in AD patients at the serine threonine‐proline positions whether by increasing the activity of glycogen synthase kinase (GSK) and protein kinase A along with cyclin‐dependent kinase 5.^43^, ^44^ Kaur et al.^45^ and Hur et al.^46^ previously highlighted that HSV‐1 infection has the ability to disturb amyloid precursor protein (APP) leading to the accumulation of the neurotoxic Aβ aggregates and oligomers leading to cognitive decline among infected subjects.

### 5.1 Clinical and postmortem findings

Several studies demonstrated the repeated presence of HSV‐1 infection in postmortem brain tissues findings detected by polymerase chain reaction (PCR) and in situ hybridization.^47^ HSV‐1 was observed to be identified in different brain regions including the cerebral cortex, and cerebellum.^48^ These findings are in accordance with the rapid spread and localization of HSV‐1 from the peripheral nervous system (PNS) to the CNS.^35^ Additionally, 72% of infected HSV‐1 subjects were associated with β‐amyloid plaques deposition in AD patients when compared to healthy control subjects. This indicates that HSV‐1 may act as a predisposing risk factor for AD pathology.^49^, ^50^, ^51^ Whereas, it was previously reported that the reactivation of HSV‐1 infection leads to elevated Aβ‐depositions in the brain associated with a relevant decrease in Aβ plasma level.^35^, ^52^, ^53^ These studies demonstrated that HSV‐1 infection results in AD‐like pathology one‐week postinfection as a drawback of successive neuronal loss, reactive gliosis, and neuroinflammation.^37^ The role of viral HSV‐1 infection in the activation of M1 microglia type has been repeatedly studied in the context of cognitive dysfunctions related to HSV‐1 infection.^17^ Whereas, HSV‐1 activates M1 microglia phenotype and astrocytes whether following penetrating the BBB or mediated by severe inflammation in the peripheral circulation producing elevated levels of pro‐inflammatory cytokines such as IL‐6.^17^, ^54^ These findings suggest that microglia cells can be considered key mediators in the neuroinflammatory related to HSV‐1 brain infection.^55^, ^56^, ^57^ The postmortem findings of^50^, ^58^ demonstrated that Aβ plaques were detected in the brain of AD patients with HSV‐1 infection. Additionally, it was reported that HSV‐1 infection induces Aβ plaques depositions intracellularly and extracellularly via different molecular mechanisms.^59^ Previous postmortem studies reported different brain regions infected with HSV‐1 associated with detected Aβ and tau protein depositions.^60^ Meanwhile, another study conducted by Deatly et al.^61^ reported that 17 AD patients out of a total of 21 were found to be infected with HSV‐1 in the trigeminal nerve. Whereas, Jamieson et al.^62^ indicated in their findings that PCR test has detected positive HSV‐1 infection in different brain regions including the hippocampus in 14 AD patients out of 21. It was also demonstrated that HSV‐1 may not only act as an instant active causative agent by itself but may also play a major predisposing factor for AD pathogenesis through various interactions with the host immune system resulting in instant acute infection or remain silent as a latent phase.^49^, ^63^ Another study also reported that both HSV‐1 and COVID‐19 viral infections may induce Aβ protein deposition in the human CSF along with a relevant observed reduction in the gray matter thickness and whole brain volume.^64^ Similar previous findings have demonstrated that the reactivation of HSV‐1 viral infection was shown to increase cognitive dysfunctions and associated AD risks.^65^

## 6 EXTRACELLULAR VESICLES (EVS) AND ALZHEIMER'S DISEASE

Extracellular vesicles (EVs) are recognized as nanosized vesicles containing lipid bilayer and were found to be secreted by all cell types.^66^ They may act as a protector and shuttle‐transfer carriers. Moreover, EVs have the ability to cross the BBB.^67^ Thus it was previously reported that EVs can contribute to the spreading of Aβ and tau proteins to different brain regions leading to AD.^67^, ^68^, ^69^, ^70^ However, other studies have highlighted the neuroprotective effects and the therapeutic approaches of EVs in hindering and alleviating AD.^71^ It was previously reported that EVs may act as a pathogenic factor for viral infections. EVs can carry viral proteins, nucleic acid, and particles such as spike protein to protect them from instant antibody neutralization.^67^, ^72^ With EVs help, the viruses attempt to be more aggressive with the ability to manipulate the immune responses. Thus it is suggested that EVs can act directly or indirectly in mediating viral infection hypothesis leading to severe infections and uncommon complications including neurodegenerative diseases. It was previously reported that HSV‐1 and VZV may use EVs to penetrate the BBB leading to severe neural infection, triggered cytokine storms, and dementia.^67^ On the other hand. EVs can indirectly result in neuroinflammation and dementia as it is suggested that EVs may carry various pro‐inflammatory mediators such as chemokines and cytokines produced as a drawback of peripheral HSV‐1 or VZV viral infection. Since EVs can easily cross the BBB, they may transmit these carried pro‐inflammatory signals from the periphery circulation to the brain leading to severe neuroinflammation and AD pathology in the long term^73^, ^74^, ^75^ as illustrated in Figure 2.

## 7 FUTURE DIRECTIONS AND CONCLUSION

Infections mainly caused by HSV‐1 and VZV may contribute to the pathogenesis of AD as a drawback of chronic neuroinflammation. It can be related to the triggered pro‐inflammatory mediators promoting Aβ plaques aggregations, and hyperphosphorylated tau protein depositions in the brain. Viral infection may be considered a key pathogenic predisposing factor in the development and the degree of AD severity as previously evidenced in the current review. On the other hand, EVs are considered valuable tools for intercellular communication in healthy individuals. Their ability to be highly manipulated by viral infections raises several questions about their role in viral penetration into the BBB leading to neuroinflammation and AD pathology. Infected patients are advised to be monitored routinely post infections to avoid any irreversible long‐term brain damage. Meanwhile, the used types of antiviral drugs against AD are required to be carefully chosen, especially if given in combination with anti‐inflammatory drugs. This also requires strict monitoring of the duration of treatment and different stages at which their usage would be most effective. Even if their effects only hinder the symptoms and AD progression, this will enormously have beneficial effects on the quality of patients' life. Moreover, the therapeutic effectiveness of the anti‐VZV vaccination against HSV‐1 should be addressed and studied in randomized trials with a wide cohort of patients. These studies are required to extend for many years to find out the exact outcomes. Thus, further preclinical and clinical studies are highly required to define the impact of viral infections and the role of EVs on AD. This will allow researchers to find out novel anti‐infectious drugs or compounds and to determine the estimated time window application of the antiviral intervention as a promising future clinical protocol for AD.

## AUTHOR CONTRIBUTIONS

Mai M. Anwar organized the main ideas of the current review, finalized them, and submitted the final version for review.

## CONFLICT OF INTEREST STATEMENT

The author declares no conflict of interest.

## ETHICS STATEMENT

Not applicable.

## TRANSPARENCY STATEMENT

The author confirms that this manuscript is honest and transparent. I declare that no important aspects of the study have been omitted and that any discrepancies from the study have been explained.

## Data Availability

Data used in the study are available upon reasonable request.
